# Supplementary material for: COVID-19 and Respiratory Virus Co-Infections: A Systematic Review of the Literature
Source: Viruses. 2023 Mar 28;15(4):865. doi: 10.3390/v15040865 (PMC10142898; doi:10.3390/v15040865)
Supplement: Supplementary file 1 [file viruses-15-00865-s001.zip › viruses-2237434-supplementary.pdf]

Supplementary File S1. Articles reporting SARS-CoV-2 and influenza virus co-infections (as of April 12, 2022).

Study / country	Time period	Number of co-infected cases / Characteristics	Findings	Management / Outcome
Kondo et al [9] /Japan	2020	57-year-old male with COVID-19 and influenza<br><br>Comorbidities: DM, vasospastic angina	1st fever and cough<br>2nd SOB, anosmia, ageusia, fever<br>1st CT: GGO in the left lung<br>2nd CT: peripheral, bilateral, GGO with consodilation and intralobular lines	1st garenoxacin<br>2nd azithromycin, meropenem, favipiravir<br>Hospitalization: yes<br>Oxygen: yes<br>Intubation: no<br>Discharge: yes
Khodamoradi et al [10] /Iran	2020	4 cases (30% of 12 COVID-19 patients)<br>1 female (74 years)<br>3 males (40, 64, 50 years)<br>Comorbidities: 1.ischemic cerebrovascular accident, HTN	Fever (4), dyspnea (4), dry cough (3), malaise (2), body pain (2), headache (3), orthopnea (2), anorexia cough, sweating, chills, severe compressive chest pain, diarrhea, respiratory distress<br>CXR: bilateral infiltrates (4)<br>Influenza A (4)	NA<br>Hospitalization: 4<br>Oxygen: NA<br>Intubation: NA<br>Unknown outcome: 4
Hashemi et al [11] / Iran	2020	23 cases (22.3% of 105 dead COVID-19 patients)	Influenza A: 18 cases (78.3%)<br>Influenza B: 5 cases (21.7%)<br>Also, RSV and bocavirus in 9.7% of SARS-CoV-2 positive dead cases, parainfluenza viruses in 3.9%, hMPV in 2.9%, and adenovirus in 1.9%.	Death: 23(100%)
Huang et al [12] / Taiwan	2020	One 48-y.o. woman<br><br>No comorbidities	Fever, dry cough, sore throat, myalgia, fatigue<br>CT: clear at admission<br>1st CXR bilateral pneumonia patches<br><br>Influenza B	oseltamivir,<br>baloxavir<br>ceftriaxone, azithromycin<br>hydroxychloroquine<br>Hospitalization: 1<br>Oxygen: NA<br>Intubation: NA<br>Discharge: 1
Singh et al [13] /USA	2020	3 cases<br>Mean age: 59.2 years (range: 41-71 years)<br>1 male, 2 females<br><br>Comorbidities: HTN, DM	Cough, fever, SOB, myalgia<br><br>CXR: bilateral patchy infiltrates<br><br>CT: diffuse scattered GGOs, multi-lobar infiltrates<br>RADT: influenza A (1), influenza B (2)<br>PCR: SARS-CoV-2	oseltamivir (3)<br>hydroxychloroquine (3)<br>azithromycin (3) ceftriaxone (3) tocilizumab (1)<br>convalescent plasma (1)<br>Hospitalization: 3<br>Oxygen: NA<br>Intubation: 2<br>Discharge: 3
Sang et al [14] / USA	2020	One 58-y.o. woman<br>Comorbidities: RA, ILD, COPD, HTN	Fever, respiratory distress<br>Complication: ARDS, cardiomyopathy (autopsy)<br>PCR: Influenza A, SARS-CoV-2	Oseltamivir, antibiotics<br>Hospitalization: 1<br>Oxygen: 1<br>Intubation: 1<br>Death: 1

Swets et al [15] / UK	February 2020-December 2021	583 (8.4%) of 6965 SARS-CoV-2 patients with viral co-infection: 227 influenza, 220 RSV, and 136 adenoviruses	NA	Hospitalization: 583<br>Intubation: NA<br>Oxygen: NA<br>Unknown outcome: 583<br>Influenza co-infection: increased risk of intubation and death
Liu et al [25] / China	2019-2020	Hospitalized children with LRTIs tested for RSV, adenovirus, parainfluenza 1,2,3, influenza A and B, hMPV, rhinovirus<br><br>7107 patients (4600 in 2019 and 2507 in 2020)<br><br>Median age: 14 months in 2019 vs 2 months in 2020	2019 vs 2020: RSV (6.6% vs 4.5%), adenovirus (2.9% vs 0.2%), influenza A (1.3% vs 0.4%), influenza B (0.7% vs 0.2%0, parainfluenza 1 (0.8% vs 0.2%), parainfluenza 3 (3.8% vs 2.1%0, hMPV (0.6% vs 0.2%), rhinovirus (7.4% vs 11%).<br>Overall positivity: 23.3% in 2019 vs 18.3% in 2020	NA<br><br>Length of stay: median 7 vs 9 days (<0.001).<br>No difference in ICU admission and mortality
Fujita et al [27] / Brazil	2021-2022	Rates of SARS-CoV-2 and influenza co-infection: Sao Paolo: 110/205 (53.6%); Bahia: 11/185 (5.9%); Pernambuco: 31/179 (17.3%); Parana: 0/75 (0%); Rio de Janeiro: 2/4,752 (0%); Amazonas: 0/494 (0%)	NA	NA
Yue et al [34] / Wuhan, China	January - February 2020	179 cases (57.3% of 307 COVID-19 patients)	Most patients had typical COVID-19 (fever and cough) regardless of influenza infection<br>Influenza A co-infection: lighter disease, higher lymphocytes and eosinophils, less often chest CT abnormalities<br>Influenza B co-infection: more severe disease: fatigue (13%), chest CT abnormalities: (100%), lower lymphocytes/ eosinophils<br>Influenza A: 49.8%; Influenza B: 7.5% early co-infected patients	Same treatment for all<br><br>Prognosis:<br>COVID-19 only: 7.6%<br>Influenza A co-infection: 5.9%<br>Influenza B co-infection: 30.4%
Maa et al [35] / Wuhan, China	NA	46 cases (49.5% of 93 COVID-19 patients)	Survivors: fever 73.9%, fatigue 30.4%<br>Non-survivors:fever 72.7%<br>Complications:<br>survivors: cardiac injury 86.4%	NA<br>Hospitalization: 46<br>Oxygen: NA<br>Intubation: NA<br>Discharge: 24

			non-survivors: higher WBCs, neutrophil count, creatinine levels, D-dimers, and TNFα<br>Influenza A: 44 (47.3%)<br>Influenza B: 2 (2.2%)	Death: 22
Cheng et al [36] /Beijing, China	January - March 2020	97 cases (45.5% of 213 COVID-19 patients)<br><br>Median age: 61 y.o.	All: fever, cough, dyspnea, expectoration, GI symptoms<br><br>Influenza A: 97 (100%)	Hospitalization: 97<br>Oxygen: N/A<br>Intubation: N/A<br>Discharge: 97<br>Influenza no effect on outcome
Zheng et al [37] /Wuhan, China	January 2020	4 cases<br>Mean age: 35 years (range: 15-62 years)<br><br>Comorbidities: malignancy	Fever and cough (3)<br><br>Prolonged fever (24 days), oxygen therapy (1)<br><br>H3N2: (1), B/Victoria: (3)	Oseltamivir and/or antibiotics<br>Hospitalization: 1<br>Oxygen: 1<br>Intubation: 0<br>Discharge: 1
Miatechet al [38] / USA	March – April 2020	4 cases (4.9% of 81 COVID-19 patients)<br>Mean age: 61.5 years (range: 58-66 years)<br>2 males, 2 females<br><br>Comorbidities (3): DM (3), HTN (3), obesity (3), HF (1), ESRD (1)	Fever (4), cough (3), SOB (2), myalgia (2), diarrhea (1), malaise (1), dyspnea (1)<br>CXR: infiltrate (2), mild vascular congestion (1), patchy diffuse bilateral infiltrates (1), streaky opacities bilaterally (1)<br><br>PCR: SARS-CoV-2 (4)<br>RADT: Influenza A (2) and B (2)	azithromyicin(1)<br>levofloxacin (2)<br>vancomycin (2)<br>metronidazole(1)<br>augmentin(1)<br>oseltamivir (1)<br><br>Hospitalization: 3<br>Oxygen: 1<br>Intubation: 1<br>Discharge: 3
Konala et al [39] / USA	NA	3 cases<br>Ages: 35, 57, 68 y.o.<br>1 male, 2 females<br><br>Comorbidities in 2 cases: DM (2), HTN (2), MI (1), CKD (1)	Fever (2), myalgia (1), cough (2), SOB (3) headache (2), sore throat (1), respiratory distress (2), diarrhea (2), altered mental status (1)<br>CXR: bilateral alveolar infiltrates (1), bilateral pleural effusions (1), air splace opacities bilaterally (1), congestion (1)<br>CT: patchy bilateral GGOs (1), bilateral scattered infiltrates (2)<br>Influenza A: 3	azithromycin (3)<br>oseltamivir (2)<br>ceftriaxone (3)<br>hydroxychloroquine (3)<br>Hospitalization: 3<br>Oxygen: 1<br>Intubation: 1<br>Discharge: 2<br>Death: 1
Ding et al [40] / Wuhan, China	NA	5 cases (4.3% of 115 COVID-19 patients)<br>Mean age: 50.2 years (range: 39-66 years)<br>2 males, 3 females<br><br>Comorbidities in 5 cases: HTN (2), CVD (1), Ca (1)	Fever (5), cough (5), SOB (5), myalgias (2), fatigue (2), headache (2), expectoration (2), pharyngeal pain (3)<br><br>Complications: ARDS (1), acute liver injury (3)<br>Remission of CT findings<br>Mean hospitalization: 25.6 days	antibiotics (5)<br>oseltamivir (5)<br>other antivirals (5)<br>glycosteroids(3)<br>Hospitalization: 5<br>Oxygen: 5<br>Intubation: 0<br>Discharge: 5

			(range: 16-39 days)	
Wu et al [41] /Beijing, China	January 2020	One 69 y.o. case<br><br>Comorbidities: (-)	Severe hypoxemia<br>Complications: ARDS<br><br>CT: bilateral, mass, GGOs<br>CXR: diffuse exudative shadows in bilateral lungs<br><br>BAL: positive for SARS-CoV-2<br>Influenza A	oseltamivir (1)<br><br>Hospitalization: 1<br>Oxygen: 1<br>Intubation: 1<br>Discharge: 1
Xiang et al [42] / Wuhan, China	NA	2 cases (1.4 % of 145 COVID-19 patients)<br>1 male 41 y.o., 1 female 34 y.o.<br><br>Comorbidities in 1 case	Fever (1), cough (2), dyspnea (1), myalgia (2), fatigue (2)<br><br>Complications: hypoxemia (1)<br><br>CXR: abnormal (2)	oseltamivir (2)<br>other antivirals (2)<br>antibiotics (2)<br><br>Hospitalization: 2<br>Oxygen: 1<br>Intubation: 0<br>Discharge: 2
Cuadrado-Payán et al [43] / Spain	NA	4 cases<br>Mean age: 67 years (range: 53-81 years)<br>3 males, 1 female<br><br>Comorbidities: HTN (4), DM (2), CKD (2)	Fever (4), dyspnea (4), non-productive cough (4), bronchospasm (3), low oxygen saturation (3)<br><br>CXR: bilateral infiltrates (1), right lobal pneumonia (1),normal(2)<br>Influenza A: (2)<br>Influenza B: (1)<br>Influenza A+B: (1)	oseltamivir (4)<br>hydroxychloroquine (4)<br>lopinavir-ritonavir (4)<br><br>Hospitalization: 4<br>Oxygen: 3<br>Intubation: 3<br>Discharge: 1<br>Unknown outcome: 3
Konala et al [44] / USA	Early 2020	One 66-yo woman<br><br>Comorbidities: obesity, ischemic cardiomyopathy, DM, HTN, CAD, CKD	syncoptic episode, fever, SOB, non-productive cough, decreased appetite, hypoxia<br><br>CXR: right lower lobe infiltrate<br><br>Low WBCs and lymphocytes<br><br>Influenza A	azithromycin, oseltamivir, ceftriaxone, hydroxychloroquine<br><br>Hospitalization: 1<br>Oxygen: 1<br>Intubation: 1<br>Unknown outcome: 1
Hashemi et al [45] /Iran	March-April 2020	2 cases<br><br>78 y.o. female<br>75 y.o. male<br><br>Comorbidities: chronic lung disease (1)	Fever (2), dyspnea (2), joint pain (2), cough (1), headache (1), confusion (1), joints pain (2), nausea (1), diarrhea (1)<br><br>Increased WBCs (1), low WBCs (1), increased CRP (2), lymphopenia (2)<br>Complications: ARDS (1)<br>CT: peripheral GGOs (2), interalobular septal thickening (2)	Hydroxychloroquine (2)<br>lopinavir/ritonavir (2)<br>Hospitalization: 2<br>Oxygen: 2<br>Intubation: 2<br>Death: 2

			Influenza A (2)	
Munivenkatappa et al [46] / India	April 2020	One 65-y.o. male<br><br>Comorbidities: obesity, COPD, DM, CKD, HTN, hypothyroidism	Breathlessness, productive cough, no fever, oxygen saturation <90, normal WBCs, lymphopenia<br><br>Complications: ARDS<br><br>CXR: bilateral patchy consodilation<br>PCR: Influenza A, SARS-CoV-2	Oseltamivir, ceftriaxone, azithromycin, hydroxychloroquine<br><br>Hospitalization: 1<br>Oxygen: 1<br>Intubation: 1<br>Death: 1
Coutinho et al [47] / UK	2020	One 74-y.o. male<br><br>Comorbidities: COPD, DM, HTN	Non-productive cough, low-grade fever, myalgia, malaise. SOB, 86% oxygen saturation<br><br>Lymphopenia, increased CRP<br><br>CXR: GGOs bilaterally<br>PCR: influenza B, SARS-CoV-2	oseltamivir, hydrocortizone, doxycycline, azithromycin<br><br>Hospitalization: 1<br>Oxygen: 1<br>Intubation: 1<br>Discharge: 1
Azekawaa et al [48] / Japan	2020	One 78-y.o. woman<br><br>Comorbidities: hypothyroidism, dislipidemia	Mmalaise, anorexia, cough, low-grade fever<br><br>CXR: bilateral reticular shadow<br>CT: GGO<br><br>Influenza A	oseltanivir<br>ceftriaxone<br>azithromycin<br><br>Hospitalization: 1<br>Oxygen: 0<br>Intubation: 0<br>Discharge: 1
Yu et al [49] / China	NA	64 co-infected cases (50% of 128 COVID-19 patients)	Ccoinfected patients vs non-coinfected patients:<br>1.no difference in lung involvement, 2.longer median virus shedding from admission for coinfected patients, 3.no relation with pneumonia severity<br><br>lopinavir/ritonavir treated co-infected patients: faster pneumonia resolution within 2 weeks (37% vs 1%, p=0.001)<br><br>Influenza A: 54 (84.4%)<br>Influenza B: 10 (15.6%)	lopinavir/ritonavir N/A<br><br>Hospitalization: 64<br>Intubation: N/A<br>Oxygen: N/A<br>Discharge: 64
D'Abramo et al [50] / Italy	2020	One 56-y.o. male<br><br>Comorbidities: smoker, overweight, MI	Fever, diarrhea, asthenia lymphopenia, increased CRP and serum fibrinogen levels<br>1st CT: bilateral multiple peripheral GGOs<br>2nd CT: worsening bilateral	oOseltamivir lopinavir/ritonav ceftriaxone, azithromycin, methylprednisolone iv<br><br>Hospitalization: 1<br>Oxygen: 1

			GGO, bilateral fibrotic consodilation<br>Complications: respiratory failure<br>PCR: Influenza A, SARS-CoV-2	Intubation: 0<br>Discharge: 1
Lozano-Parras et al [51] / NA	January 2020	46-y.o. woman<br><br>Comorbidities: RA, ILD, COPD, HTN	Dry cough, fever, prostration, arthromyalgia, dyspnea<br>89% oxygen saturation.<br>Low WBCs, lymphopenia, low platelets<br>CXR: consolidation in left upper lobe and right perihilar region<br>PCR: Influenza A, SARS-CoV-2	oseltamivir, levofloxacin<br>systemic steroids<br><br>Hospitalization: 1<br>Oxygen: 1<br>Intubation: 0<br>Discharge: 1
Jing et al [52] /USA	2020	60-y.o. woman<br><br>Comorbidities: ESRD	Non-productive cough, SOB, diarrhea, fever, worsening influenza-like symptoms, abdominal pain, nausea, vomiting<br><br>CT: multifocal areas of consodilation, extensive hilar and mediastinal adenopathy<br><br>RADT: Influenza A<br>PCR: SARS-CoV-2	oseltamivir<br>vancomycin/cefepime<br><br>Hospitalization: 1<br>Oxygen: N/A<br>Intubation: N/A<br>Discharge: 1
Lew et al [53] / USA	2020	32-y.o. man<br><br>No comorbidities	Fever, cough, SOB<br><br>CXR: peripheral patchy opacities in lower lung zones<br>2nd CXR: worsening opacities<br>CT: extensive bilateral GGOs with areas of confluent consodilation in posterior lung bases<br>Complications: ARDS, acute kidney injury (hemodialysis), diabetes	ceftriaxone, azithromycin, hydroxychloroquine<br>linezolid,<br>oseltamivir<br>lopinavir/ritonavir<br><br>Hospitalization: 1<br>Oxygen: 1<br>Intubation: 1<br>Death: 1
Alosaimi et al [54] / Saudi Arabia	2020	34 co-infected cases (71% of 48 COVID-19 patients)<br>Influenza A H1N1 (17 cases, 50% of co-infected), *C. pneumoniae*(13 cases), adenovirus (10 cases)	NA	Hospitalization: 48<br>Oxygen: N/A<br>Intubation: N/A<br>Death: 1/3 of no co-infected COVID-19 cases, 2/3 of co-infected cases<br><br>Association of influenza A with mortality

Fontana et al [55] / Italy	March – June 2020	152 respiratory specimens positive for SARS-CoV-2<br>(66 from BAL; 86 from SPU)<br><br>93 males, 59 females<br><br>Mean age: 65 years	93 (61%) FAPP positive for bacteria and/or viruses:<br><br>Bacteria: *S. aureus*(30/44, 62%), and Gram-negative pathogens: *P. aeruginosa*, *K. pneumoniae*, *A. baumannii*, and *H. influenzae;*<br><br>One case with influenza A	NA
Maddali et al [56] / USA	N/A	two-week-old healthy-term female neonate	apnea, nasal congestion, no fever, no cough, no vomiting<br>100% oxygen saturation<br>CXR: no consodilation<br>RADT: Influenza<br>PCR: SARS-CoV-2	NA<br><br>Hospitalization: 1<br>Oxygen: 0<br>Intubation: 0<br>Discharge: 1
Aggarwal et al [57] / India	July 2021 - January 2022	13,467 samples from both community and hospital settings tested for SARS-CoV-2/influenza virus co-infection during ILI/SARI sentinel surveillance	5 (0.04%) with SARS-CoV-2/influenza virus co-infection	NA<br><br>Hospitalization: 3/5 co-infecte[illegible] patients<br>Oxygen: 0<br>Intubation: 0<br>Discharge: 3
Alhoufie et al [58] / Saudi Arabia	May - June 2020	1st case: 66 y.o. female<br>2nd case: 44 y.o. male<br><br>Comorbidities<br>1. DM type 2<br>2. no comorbidities	Fever (2), productive cough (2), chest pain (1), SOB (2), diarrhea (1),<br>88% oxygen saturation (1)<br><br>CXR: 1.bilateral coarse reticular shadow, 2.bilateral hilar bronchial wall thickening<br><br>Also, parainfluenza type 2<br>Influenza IgM and Influenza B	1. ceftriaxone, hydroxychloroquine, methylprednisolone<br>2. amoxicillin-clavulanate, azithromycin, hydroxychloroquine<br><br>Hospitalization: 2<br>Oxygen: 1<br>Intubation: 0<br>Discharge: 2
Ishiguro et al [59] / Japan	February 2020 - January 2021	298 patients (mean age: 61.1 y.o.) with CAP due to severe SARS-CoV-2 infection<br><br>Comorbidities: in 63.4% of patients	Primary viral pneumonia: 90.3%, mixed viral and bacterial pneumonia: 9.7% (mainly *M. pneumoniae*)<br>Viral coinfection in 30.5% of patients with viral pneumonia. Influenza and parainfluenza viruses were the most common co-viruses (9.4% and 9.1%), coronavirus (6.0%), adenovirus (4.7%), bocavirus (3.4%), rhinovirus (3.0%), parechovirus(2.3%), hMPV (2.0%), RSV (2%), enterovirus (1.3%).	Mortality: 7.7%<br><br>Viral and bacterial co-infections were not associated with severity on admission, need for high-flow nasal cannula, invasive ventilation, and mortality.

Rizzo et al [60] / USA	September 2020 - April 2021	Of 255 influenza-positive patients also tested for SARS-CoV-2, 58 (23%) positive.<br>22 females (38%), 35 males (60%), unknown 1 (2%)<br>Age groups: 5 (9%) 0-17 y.o., 23 (40%) 18-49 y.o., 12 (21%) 50-64 y.o., 18 (31%)>65 y.o.	Among co-infected persons: 39 (67%) had influenza B.<br>Among 28 with hospitalization record, 11 (39%) were hospitalized. Five (9%) died, including two who were hospitalized. All five who died were >50 years old.	Hospitalization: 11<br>Intubation: N/A<br>Oxygen: N/A<br>Discharge: 9<br>Death: 2 and 3 non-hospitalize
Heshmat-Ghahdarijani et al [61] / Iran	February – March 2020	Of 1639 SARS-CoV-2 patients only 2 were positive for influenza B.<br><br>76 y.o. male, 62 y.o. female<br><br>Comorbidities: DM, HTN, CHD, CKD	Fever (2), dyspnea (1), body pain (1), dry cough (1), hemoptysis (1), muscular pain (1), respiratory distress (1)<br><br>CXR: bilateral diffuse infiltrates<br><br>CT: bilateral multiple patchy GGOs<br><br>Influenza B and SARS-CoV-2	levofloxacin iv (1)<br>oseltamivir p.o.(2)<br>hydroxychloroquine (2)<br>methylprednisolone i.v.<br>ceftriaxone i.v(1)<br><br>Hospitalization: 2<br>Oxygen: N/A<br>Intubation: N/A<br>Discharge: 2<br>Both cases symptom- free after 3-months follow-up
Ali et al [62] / USA	A	Five co-infected cases (61 y.o. female, 33 y.o. male, 46 y.o. male, 57 y.o. female, 41 y.o. male)<br><br>Comorbidities: asthma, fever, COPD	Cough (3), fever (5), diarrhea (1), SOB (3), low oxygen saturation (2), double vision, myalgias (2), vomiting (1)<br>CXR: diffuse patchy bilateral infiltrates, patchy left lower lung density, mild diffuse bronchial wall thickening, extensive GGOs at peripheral zones<br>CT: bilateral multi-lobar peripheral GGOs<br>raised inflammatory markers (5)<br>PCR/RADT: SARS-CoV-2 (5)<br>RADT: Influenza A (1)<br>RADT: Influenza B (3)<br>PCR: Influenza B (1)	remdesivir (5)<br>oseltamivir (4)<br>azithromycin (1)<br>dexamethazone(4)<br>ceftriaxone (1)<br>metronidazole (1)<br><br>Hospitalization: 5<br>Oxygen: 1<br>Intubation: 1<br>Discharge: 5
Zheng et al [63] / China	January - November 2020	220 adults with H1N1, 285 with COVID-19, 36 with COVID-19 and influenza H1N1<br>Median age of COVID-19 patients: 47 y.o.<br>Median age of H1N1 patients: 50 y.o.	Coinfected patients had more fever and myalgia than COVID-19 patients	SARS-CoV-2 and H1N1 co-infection more severe disease c/w mono-infection with influenza or SARS-CoV-2<br>SARS-CoV-2 vs H1N1 co-infection: 2.8% mortality vs 0[illegible] of COVID-19 patients; 13.9% ICU admission vs 5.2%

Akhtar et al [64] / Bangladesh	Marchto December 2020	1986 patients with SARI (median age: 28 years):<br>285 (14.3%) SARS-CoV-2-positive patients, 175 (8.8%) influenza-positive patients, 5 (0.3%) SARS-CoV-2 and influenza coinfected patients	NA	31 (10%) patients with SARS-CoV-2 SARI died<br><br>One-month post-discharge: 25 additional deaths (9.1%) in SARS-CoV-2 infected patients<br>No in-hospital or post-discharge mortality within one month among the 5 coinfected patients.
Venkatram et al [65] / USA	2017-2021	412 influenza patients vs 18 co-infected patients<br>Mean age: 60 y.o.<br>Comorbidities: common	NA	NA<br><br>Overall mortality: 6.5%.<br>COVID-19: no increased mortality among co-infected patients
Sreenath K, et al. [66] / India	June 2020-January 2021	Of 191 COVID-19 patients, 89 (46.6%) had co-infection with ≥1 pathogens<br>Median age: 50 years<br>Comorbidities: 140 (89%)	Overall, 13 co-pathogens were identified:<br>Bacterial coinfections: 41.4%<br>Viral co-infections: 7.3% (mostly adenovirus and rhinoviruses)	NA
Jeong et al [67] / Korea	August 2020 - October 2021	35/436 adult COVID-19 patients (57 inpatients and 379 outpatients) with influenza co-infection<br>Coinfected adults; older median age (70 vs 46 years); more often admitted (85.7% vs 6.7%)	Coinfected patients had significantly higher Cts and inflammatory markers, but less lymphocyte count	Risk factors for mortality: co-infection, older age, elevated lactate dehydrogenase
Castillo et al [68] /USA	March 2020	588 patients tested for SARS-CoV-2 with other respiratory infections on presentation	51 (8.7%) patients were positive for COVID-19 with one positive for another respiratory infection and one for influenza A	NA

SARS-CoV-2: severe acute respiratory syndrome coronavirus 2; COVID-19: coronavirus disease 2019; SOB: shortness of breath; DM: diabetes mellitus; CT: computer tomography; GGOs: ground glass opacities; PCR: polymerase chain reaction; NA: not available; HTN: hypertension; CXR: chest-X-ray; USA: United States of America; RADT: rapid antigen detection test; RA: rheumatoid arthritis; ILD: interstitial lung disease; ARDS: acute respiratory distress syndrome; COPD: chronic obstructive pulmonary disease; UK: United Kingdom; RSV: respiratory syncytial virus; vs: versus; LRTIs: lower respiratory tract infections; hMPV: human metapneumovirus; ICU: intensive care unit; WBCs: white blood cells; TNFα: tumor necrosis factor α; GI: gastrointestinal tract; HF: heart failure; CKD: chronic kidney disease; ESRD: end-stage renal disease; MI: myocardial infarction; CRP: C-reactive protein; CVD: cardiovascular disease; Ca: cancer; BAL: bronchoalveolar lavage fluid; CAD: coronary artery disease; CFU: colony forming units; SPU: sputum; *S. aureus: Staphylococcus aureus*; *P. aeruginosa: Pseudomonas aeruginosa; K. pneumoniae: Klebsiella pneumoniae; A. baumannii: Acinetobacter baumannii; H. influenzae*: *Haemophilus influenzae;* TOF MS: time of flight mass spectrometry; ILI/SARI: influenza-type illnesses/severe acute respiratory infection; CAP: community-acquired pneumonia; *M. pneumoniae*: *Mycoplasma pneumoniae*; CHD: congestive heart disease; c/w: compared with; Ct: cycle threshold value

Supplementary File S2. Articles reporting co-infections of SARS-CoV-2 and respiratory viruses other than influenza (as of April 12, 2022).

Study / country	Time period	Number of co-infected patients / Characteristics	Findings	Management / Outcome
Uhteg et al [18] / USA	December 2019-October 2021	4/173 SARS-CoV-2 positive samples were also positive for enterovirus/rhinovirus (3) or adenovirus (1)<br>Overall, enterovirus was the prevalent co-virus<br>Coinfected patients were mostly children (≤18 y.o.)	NA	NA
Kanji et al [22] / Canada	June 2020-May 2021	4818 SARS-CoV-2 positive specimens (94.8% from adults, 53% males, median age: 56 years, ~50% outpatients) tested by multiplex PCR<br>Co-infections in 134 (2.8%) cases (in children (10% vs 2.4% in adults)<br>Enterovirus (118 cases; 88.1%), adenovirus (5 cases; 3.7%), RSV (4 cases; 3%), human coronaviruses (3 cases; 2.2%), parainfluenza viruses (2 cases; 1.5%), hMPV (2 cases; 1.5%)<br>Enterovirus and adenovirus predominated in children	NA	NA
De Francesco et al [23] / Italy	January 2017 - February 2020 c/w March 2020 - May 2021	Hospitalized adults with respiratory symptoms: 10,121 samples before COVID-19 pandemic and 2362 during pandemic: positivity: 14.6% vs 2.7%<br>Before the pandemic: co-infections with different respiratory viruses (influenza A-B, hMPV, parainfluenza, RSV, coronaviruses, adenovirus)<br>During the pandemic: coinfections only with SARS-CoV-2 and rhinovirus (6 cases)	SOB, sore throat, cough and fever ≥37,5°C	NA
Ippolito et al [24] / Italy	November to February 2018/2019, 2019/2020, 2020/2021	178 hospitalized children ≤2 y.o. with LRTIs (66 in 2018/2019, 96 in 2019/2020, 16 in 2020/2021); in 171 children (96%) a virus detected (61 children (36%) with co-infections)<br>Median age: 5 (2-13) months<br>53% males<br>In COVID-19 pandemic 2/16 children positive for SARS-CoV-2: both had co-infection (bocavirus and rhinovirus)	NA	NA
Wanga et al [74] / USA	July – August 2021	113/713 (15.8%) children <18 y.o. hospitalized with COVID-19 had a viral coinfection (32.4% of < 1 y.o., 36.1% of	NA	Co-infected children: more frequently oxygen support than COVID-19 only (69%

		1-4 y.o.; 4.2% of 5-11 y.o., and 2.2% of 12-17 y.o.)<br>RSV: 75 cases (66.4 % of coinfected cases)		vs 51.2%). No difference in terms of ICU admission, duration of stay, ECMO required or outcome
Ishiguro et al [59] / Japan	February 2020-January 2021	91/298 (30.5%) patients with CAP due to SARS-CoV-2 with viral co-infection: influenza (28 cases; 9.4%), parainfluenza (27 cases; 9.1%), cold coronavirus (18 cases; 6%), adenovirus (14 cases; 4.7%), bocavirus (10 cases; 3.4%), rhinovirus (9 cases; 3%), parechovirus (7 cases; 2.3%), hMPV (6 cases; 2%), RSV (6 cases, 2%), enterovirus (4 cases; 1.3%)<br>Bacterial co-infections: 10 cases (9.7%)	NA	Co-infection was not a risk factor for need of high-flow oxygen therapy, invasive mechanical ventilation or mortality
Sreenath et al [66] / India	June 2020-January 2021	89/191 (47.1%) SARS-CoV-2 positive patients (median age: 50 years, 895 with comorbidities) had coinfection:<br>79 (41.4%) bacterial co-infection<br>14 (7.3%) viral co-infection (coronaviruses: 4, rhinovirus: 4, adenovirus: 3 cases, influenza B: 2)	Fever (88.4%), cough (60,2%), SOB (58.6%), confusion (18.8%), abdominal pain-diarrhea(9.9%), headache (7.3%)<br>Coinfected patients: more often abnormal creatinine levels, lower platelet counts, and higher CRP	Higher rates of ICU admission among coinfected patients<br><br>36 (40%) co-infected patients died
Jeong et al [67] / Korea	August 2020-October 2021	35/436 COVID-19 patients had co-infection:<br>6/436 (1.4%) with respiratory viruses: RSV (2), rhinovirus (2), bocavirus (1), influenza B (1)<br>3 cases: 20-39 y.o.<br>Comorbidities: 4 patients (HTN)	Fever (6), dyspnea (6), cough (6)<br>Chest X-ray: infiltrates (2)<br>Complications: renal failure (1)	Hospitalization: 2<br>Oxygen: 0<br>Intubation: 1<br>Discharge: 1<br>Death: 1
Chekuri et al [75] / USA	11 March-11 April 2020	14/306 (4.6%) SARS-CoV-2 positive adults (median age: 56 years) were positive for a non-influenza virus: human coronavirus (7; 46.6%), human rhinovirus (4; 26.7%), parainfluenza 3 (2; 13.3%), adenovirus (1; 6.7%), RSV B (1; 6.7%)<br>Comorbidities: 60.1% HTN, 36% DM	NA	C/w co-infected patients, those with SARS-CoV-2 only were more likely to be admitted in ICU (73.6% vs 42.9%)<br>Severe outcome in 111 (36.3%)SARS-CoV-2 infected patients vs 3 (21.4%) in co-infected patients
Soto et al [76] / Peru	September - December 2020	3/93 hospitalized patients with COVID-19 pneumonia had virus co-infection: rhinovirus (2), adenovirus (1)	NA	Unfavorable outcome: 1

Cooksey et al [77] / USA	May 2020 June 2021	23/1373 (1.7%) persons positive for SARS-CoV-2 had co-infections: 19 rhinovirus/enterovirus, 1 adenovirus, 1 *M. pneumoniae*, 1 parainfluenzae type 4, 1 human coronavirus plus parainfluenzae type 1	NA .	NA
Perez-Lazo et al [78] / Peru	July-November 2020	154 of 295 adults (52.2%) with moderate/severe COVID-19 pneumonia At admission: co-infection with *M. pneumoniae*(28.1%), *C. pneumoniae*(8.8%), both bacteria (11.5%), adenovirus (1.7%), *M. pneumoniae*/ adenovirus (0.7%), *C. pneumoniae*/adenovirus (0.7%), RSV/*C. pneumoniae*(0.3%), *M. pneumoniae*/*C. pneumoniae*/adenovirus (0.3%)	Coinfected patients more often sepsis c/w mono-infected patients (33.1% vs 20.6%), more mean duration in ICU (16 vs 8 days), and mean duration of intubation (16 vs 9 days)	No difference in mortality between co-infected and mono-infected SARS-CoV-2 patients (20.8% versus 19.2%)
Karaaslan et al [79] / Turkey	March 2020-May 2020	7/93 children (7,5%) with SARS-CoV-2 were co-infected.<br>Rhinovirus/enterovirus: 2<br>Coronavirus NL63: 2<br>Adenovirus: 1<br>*M. pneumoniae*: 1<br>Rhinovirus/enterovirus/adenovirus: 1<br>Mean age: 10,02 years (range: 1 month-17,8 years)<br>51,6% males	Fever (68.8%), cough (57%), headache (10.8%) myalgia (5.4%), also sore throat, SOB, diarrhea, abdominal pain	Significantly longer hospita stays in patients with co-infection
Ling Ma et al [80] / Wuhan, China	January 19 toFeb 26, 2020	250 patients with COVID-19 (diagnosis of co-infections by PCR and imunoassays)<br>RSV: 12 (4.8%); adenovirus: 7 (2.8%); influenza A: 2 (0.8%); influenza B: 1 (0.4%); *M. pneumoniae*: 11 (4.4%); C. pneumoniae: 13 (5.2%)	Acute fever or respiratory symptoms	No significant difference in age and sex between mono-SARS-CoV-2 and coinfected group
Zhu et al [81] / China	January 22 to February 2, 2020	243/257 COVID-19 patients (mean: age 51 y.o.) had a co-infection: 81 (31.5%) viral, 236 (91.8%) bacterial, and 60 (23.3%) fungal co-infection<br>Respiratory viruses detected: rhinovirus (12 cases, 4.7 %), adenovirus (10 cases, 3.9 %), influenza B (5 cases, 1.9 %), influenza A (2 cases, 0.8 %), bocavirus (1 case, 0.4 %), hMPV (1 case, 0.4 %)		The rates of viral co-infection (6, 35.3 %), fungal co-infection (5, 29.5 %) and bacterial-fungal co-infection (5, 29.5 %) were the highest in severe/critical category) of COVID-19 cases
oschi et al [82] / France	March to April 2020	27/643 (4.2%) SARS-CoV-2 positive patients had a virus co-infection: Rhinovirus (11), coronaviruses (5), influenza (4), parainfluenza (3), enterovirus (2), bocavirus (2),	NA	NA

		adenovirus (1), hMPV (1)<br>13 (48.2%) males<br>Mean age: 59.6 y.o.		
Allou et al [83] / France	March 18 to April 15, 2020	5/36 (13.9%) patients with COVID-19 and hypoxemic pneumonia were co-infected, 3/5 with virus: influenza H1N1 (1), coronavirus (1), rhinovirus (1)	No differences in disease severity on chest CT (mainly GGOs and bilateral infiltrates) and in hospital length of stay between co-infected and no co-infected patients	Hospitalization: 3<br>Oxygen: NA<br>Intubation: NA<br>Discharge: 3
Tang et al [84] / China	January 28 to March 15, 2020	11/78 (14.1%) patients with COVID-19 pneumonia (6 men, mean age: 42.7 years) co-infected: *M. pnuemoniae*(5; 45.5%), RSV (4; 36.4%), influenza B (1), adenovirus (1),<br>Mycoplasma and Chlamydia (1), Mycoplasma and RSV (1), C. pneumoiae + Legionella (pneumophila).<br><br>Comorbidities: CVD (1), HTN and DM (1)	Higher procalcitonin levels among coinfected patients.<br>No differences in other tests.<br><br>Almost all coinfected patients had unilateral or bilateral pulmonary legions, except one patient with influenza B but no CXR lesions.<br>No differences in sex , age, comorbidities, symptoms, CT findings, severity, length of stay between co-infected and mono-infected patients	Quinolones
Li Yet al [85] / China	January 21 to February 16, 2020	27/81 (33%) children with COVID-19 were co-infected (mainly *M. pneumoniae*: 20 cases; 25%).<br>Viral co-infection in 6 (7%): children: influenza B: 2 (2%), influenza A + *M. pneumoniae*, parainfluenza + M.	No difference in clinical and laboratory findings or hospital stays between co-	Hospitalization: 27<br>Oxygen: 0<br>Intubation: 1<br>Discharge: 27

		pneumoniae,<br>adenovirus + *M. pneumoniae*, RSV: 1 case each (1% each)<br><br>No comorbidities	infected and mono-infected children (only lower WBCs, neutrophil and lymphocyte counts)<br>Chest imaging of co-infected: consolidation in more cases	
Nowak et al [86] / USA	March16to April 20, 2020	36/1204 (2.9%) COVID-19 patients were coinfected:<br>other coronaviruses (1,5%), RSV (0,3%), adenovirus (0,2%), rhinovirus/enterovirus (0,7%), hMPV (0.4%), influenza A (0.1%)<br><br>Coinfected patients' mean age: 60 years; 56% female	NA	NA
Schirmer et al [87] / USA	September 29 2019 to May 31, 2020	56/3757 (1.5%) persons tested positive for SARS-CoV-2 had co-infection: rhinovirus, enterovirus (19), influenza (15), non-COVID-19 coronavirus (13), RSV (4), hMPV (3), parainfluenza (2), adenovirus (2), *C. pneumoniae*(1)	No differences of hospitalization rate, length of hospitalization, ICU admission, and mortality among COVID-19 only and co-infected patients	10/56 (18%) COVID-19 co-infected patients died (4 had rhinovirus and/or enterovirus, 2 had non-COVID-19 coronavirus, 1 influenza, 1 hMPV, 1 parainfluenza, 1 RSV)
Weissberg et al [88] / Switzerland	March 10, 2020	11/13 exposed cases (outbreak) developed COVID-19, of them 2 (index case and another case) had adenovirus co-infection	Fatigue, cough, fever, sore throat, rhinorrhea	NA
Si et al [89] / China	January 21 toFebruary 29, 2020	1 of 24 COVID-19 patients had parainfluenza virus 2 co-infection	NA	NA
Hashemi et al [90] / Iran	2020	3 SARS-CoV-2 infected dead children (2 females) 13 months, 6 yrs and 6 yrs old<br>Comorbidities: asthma (2)<br><br>PCR: also, hMPV (3)	Cough, fever, malaise, diarrhea lymphopenia (3), increased CRP (3)<br>CT: bilateral GGOs (3)	Hospitalization: 3<br>Oxygen: 0<br>Intubation: 3<br>Death: 3
Leuzinger et al [91] / Switzerland	January to March 2020	17/105 SARS-CoV-2 positive cases were coinfected: human coronavirus (5), rhinovirus (5), parainfluenza virus (3), influenza A (2), rhinovirus and	Influenza-like illness	NA

		adenovirus (1), rhinovirus, RSV and parainfluenza virus (1)		
Motta et al [92] / Colombia	2020	40-year-old man: COVID-19 + adenovirus (also *K. oxytoca*)<br><br>Comorbidities: DM	Odynophagea, dry cough, dyspnea, fever, fatigue, rachepnea, arthralgia, 82% oxygen saturation<br><br>CXR: right basal GGO<br>Chest CT: multilobar involvement<br>Complication: ARDS<br>(ICU for 12 days)	ampicillin, sulbactam, clarithroycin, hydroxychloroquine<br><br>Hospitalization: 1<br>Intubation: 1<br>Discharge: 1<br>On supplementary oxygen at home
Sanchez-Nadales et al [93] / USA	2020	22-year female with severe acute hypoxic respiratory failure<br><br>Coinfection: SARS-CoV-2 and HCoV NL63<br><br>Comorbidities: tubulointerstitial nephritis on steroids, HTN, DM	Nonproductive cough, Fever, chills, SOB<br>Complications: pulmonary embolism, acute kidney injury, pneumothorax, pneumomediastinum, multiple cardiac arrests	30 days ICU<br>Hospitalization: 1<br>Oxygen:<br>Intubation:<br>Death: 1
Nascimento et al [94] / Brasil	2020	25-year-old male physician with COVID-19 and measles (serology positive IgM)<br><br>Comorbidities: asthma	Prostration, fever, cough, odynophagia, anosmia, ageusia, face edema, rash, conjunctival hyperemia, tachypnea, desaturation	azithromycin, prednisone<br>Supplementary oxygen<br><br>Favorable outcome
Touzard-Romo et al [95] / USA	2020	57 y.o. female with SARS-CoV-2 and hMPV<br>Comorbidities: obstructive sleep apnea on continuous positive airway pressure, HTN, hyperlipidemia	Dry cough, SOB, fever<br><br>CXR: no infiltrates	Albuterol inhaler, steroids<br>Hospitalization: 1<br>Oxygen: 0<br>Intubation: 0<br>Discharge: 1

Sohal et al [96] / USA	2020	2 fatal cases with SARS-CoV-2 co-infection: a 40-year-old male with COVID 19 and parainfluenza and a 80-year-old female with COVID-10 and *Streptococcus pneumoniae*<br>Comorbidities (1): DM, HTN, asthma, paroxysmal atrial fibrillation, dementia	Fever (2), cough (1), body aches (1), headache (1), SOB (1)	Ceftriaxone (2), azithromycin (2), Hydroxychloroquine (1)<br><br>Hospitalization: 2<br>Intubation: 1<br>Death: 2
Danley et al [97] / USA	March 26, 2020	4-month-old boy coinfected: adenovirus + COVID-19<br><br>Comorbidities: CHD	Loose stools, stuffy nose, decreased oral intake, mild cough, diaphoresis, tachypnea, 91% oxygen saturation	No antibiotics<br><br>Hospitalization: 4 days<br>Oxygen: yes<br>Discharge
Jiang et al [98] / China	December 1 2019 - January 16 2020	Two children (both females, 6.5 years and 3.5 years):<br>1st SARS-CoV-2, RSV, hMPV 2nd SARS-CoV-2, hMPV, *M. pneumoniae*	Fever, cough, vomiting, swollen pharynx	oseltamivir (1), glucocorticosteroids(1), antibiotics (2)<br><br>Hospitalization: 2<br>Oxygen: 2<br>ICU admission: 1<br>Discharge: 2
Kakuya et al [99] / Japan	February 2020	5-y.o. boy with COVID-19 and hMPV<br><br>11-y.o. boy with COVID-19 and influenza A (positive antigen)<br><br>No comorbidities	Cough, fever, decreased appetite<br><br>No complications	ceftriaxone, peramivir<br>Hospitalization: 1<br>Oxygen: 0<br>Intubation: 0<br>Discharge: 1<br>Death: 0
Langerbeins et al [100] / Germany	2020	52-y.o. patient with SARS-CoV-2 and parainfluenza (Multiplex PCR)<br><br>Comorbidities: CLL, psoriasis, asthma	Cough, ageusia, hyposmia<br>CT: bilateral GGOs	hydrochloroquine, IVIG, piperacillin/tazobactam,<br><br>Admission to ICU<br>High flow oxygen via nasal cannula
Rodriguez et al [101] / USA	2020	21-year-old male with severe pneumonia due to SARS-CoV-2 and parainfluenza virus<br><br>substance abuse, smoker<br>no reported medical history	Fever, dry cough, dyspnea, myalgia, fatigue and diarrhea<br>Increased WBCs, CRP, ferritin, procalcitonin<br>CXR: bilateral opacities<br>CT: multiple bilateral opacities and bilateral GGOs	oseltamivir, azithromycin, cefriaxone, lopinavir/ritonavir, hydroxychloroquine<br><br>Hospitalization: twice<br>Oxygen: 1<br>Intubation: 0<br>Ooutcome: improvement

Alharthy et al [102] / Saudi Arabia	2020	25-year-old male admitted to ICU with acute respiratory failure due to COVID-19<br>No comorbidities<br><br>hMPV by PCR	fever, dry cough, wheezing, chest pain, 75% oxygen saturation<br><br>lymphocytopenia, increased CRP<br>CXR: bilateral interstitial pneumonia	lopinavir/ritonavir, ribavirin, interferon beta-1b, dexamethasone<br><br>Hospitalization: 1<br>Oxygen: 1<br>Intubation: 0<br>Discharge: 1
Scott et al [103] / USA	March 2020-February 2021	53/745 valid COVID-19 specimens (7,1%) positive for ≥1 pathogens:<br>Rhinovirus/enterovirus: 41.5%;<br>hMPV: 33.9%; adenovirus: 22.6%<br>No influenza A or B<br>Median age of co-infected patients: 38 years<br>53% males, 47% females<br>Coinfections were more common in children ≤9 y.o.<br>In children ≤9 y.o. mainly hMPV, adenovirus, RSV, bocavirus, and rhinovirus/enterovirus	NA	NA

SARS-CoV-2: severe acute respiratory syndrome coronavirus 2; USA: United States of America; y.o.: years old; NA: not available; PCR: polymerase chain reaction; RSV: respiratory syncytial virus; hMPV: human metapneumovirus; c/w: compared with; COVID-19: coronavirus disease 2019; vs: versus; SOB: shortness of breath; LRTIs: lower respiratory tract infections; ICU: intensive care unit; ECMO: extracorporeal membrane oxygenation; CAP: community-acquired pneumonia; CRP: C-reactive protein; HTN: hypertension; CXR: chest-X-ray; DM: diabetes mellitus; *M. pneumoniae*: *Mycoplasma pneumoniae*; *C. pneumoniae*: *Chlamydia pneumoniae*; CT: computer tomography; GGOs: ground glass opacities; CVD: cardiovascular disease; WBCs: white blood cells; *K. oxytoca*: *Klebsiella oxytoca*; ARDS: acute respiratory distress syndrome; CHD: chronic heart disease; IVIG: intravenous immune globulin; CLL: chronic lymphocytic leukemia
